# Dr. S. P. Hullihen

**Published:** 1858-01

**Authors:** 


The late DR. S. P. HULLIHEN, of Wheeling, Va.—If biography could do full justice to the life of Dr. Hullihen, a correct history of his professional career would be both instructive and interesting to his fellow-laborers in surgery, and especially grateful to his many friends; but this can scarcely be accomplished successfully within the limits prescribed by a medical journal.

Simon P. Hullihen was born in Point Township, Northumberland Co., Pennsylvania, on the 10th of December, 1810; his father was Thomas Hullihen, and his mother Rebecca Freeze, who went from New Jersey to Northumberland County when a child of seven years of age. The grandfather of Thomas Hullihen came from Ireland, and his family from that period continued to be industrious Pennsylvania farmers. Mrs. Rebecca Hullihen, the doctor's mother, retained, until a very advanced age to which she lived, a strong predilection for simple country life and habits. Dr. Hullihen was a mere child when his father died.

The early education of Dr. Hullihen was such as the best school in his mother's neighborhood afforded—the township district school; and although it did not embrace a very extended course of study, yet such as was taught there he eagerly and diligently pursued, and in every instance successfully mastered. His academical training terminated when he was under seventeen years of age, and from that period, except medically, he was a self-educated and self-made man.

During his boyhood, when only eight or nine years old, a serious and most painful accident befell him, which no doubt exercised an important influence both upon his mind and bodily health for the remainder of his life; and though he himself has sometimes told the story with a peculiar quaintness not to be forgotten by those who listened to him, he was never sufficiently explicit to permit the occurrence to be given in all its details of interest to the reader. While playing with other boys in the vicinity of a limekiln in which the fire had recently subsided, young Hullihen was pushed or fell through the opening at the top in among the unextinguished embers or heated stones at the bottom; before assistance could be obtained to draw him from his perilous situation, his feet were both terribly burned—so severely indeed, that he was confined to his bed for nearly two years, and in spite of his mother's constant and devoted care he was in danger of being permanently a cripple. When after long sufferings and severe trials of patience he was able to stand, the principal injuries being about the heels, he at first walked entirely upon his toes, a necessity which grew into habit, his feet remaining sadly deformed; but in time, and by the aid of contrivances, he was enabled to wear boots and to walk without the use of a cane. At an early period after his recovery from this suffering he made accurate plaster Paris casts of his own feet, and by sending them to the last-maker obtained his first comfortable boots as the reward of this natural genius for mechanics.

During the protracted illness of her son Mrs. Hullihen indulged the consoling thought that he would be able to acquire such impressions as would give him a religious turn of mind, and that she might see him a clergyman. With this idea the pious good mother taught her boy to read and study the Scriptures with persevering attention during the whole period of his confinement; and although her fond expectation as to his future profession was not realized, the Bible teachings then imparted were never forgotten by him.

Scarcely had Hullihen approached the age of manhood ere he evinced decided inclinations for medical pursuits—principally those belonging to practical surgery: he always had a desire to witness operations and see wounds dressed; and even at this time he purchased some dental instruments, and would benevolently extract teeth for suffering neighbors or acquaintances. His skill and kindness in this way soon became so well known that he relieved the country doctors from that troublesome and unprofitable practice for which the better class have neither much taste, time, or ability. He was ever ambitiously reading the best medical and surgical works he could get hold of, and at the period of his eventual success and widely extended fame as an operator his medical library consisted of the most reliable writers, and the volumes themselves bore evident marks of long and frequent use by their owner.

Dr. Hullihen commenced practice as a surgeon and dentist at Canton, Stark County, Ohio, in the year 1832, but there are no means of ascertaining with what amount of good or ill fortune, unless by inference from the fact that when he married Miss E. Fundenburg, at Pittsburg, in April, 1835, he immediately transferred his residence to Wheeling, Va. Here he continued until his death, in March, 1857. The settlement of a young surgeon in a western town twenty-two years ago, and one especially who dared to announce himself as intending to practice dentistry as a branch of his profession, was not likely to meet with sympathy or cordiality from the medical practitioners already established there; but, on the contrary, the petty jealousies that proverbially belong to doctors were, in this instance, more than ordinarily bitter. Yet it may justly be a matter of astonishment and disgust that these bad feelings should be fostered in the breast of even a single man, to the degree that would induce the circulation of gross and malignant slanders, with the design and hope of preventing success in a fellow practitioner. It was perhaps this element of early and undeserved persecution that greatly contributed to excite all the ambition of a noble character, and caused good to come out of evil, as witnessed by his ultimate prosperity and usefulness. It would be out of place to introduce here, however creditable as well as characteristic of Hullihen, a detailed history of the unworthy opposition he encountered and triumphed over at the onset of his professional career in Wheeling; the public records there have, years ago, placed that matter among the evidences that "truth is mighty and will prevail," and that the humble virtuous man may obtain full justice if he has the courage perseveringly to demand it. While suffering under the unkind and unjustifiable efforts to misrepresent his character and drive him from Wheeling, an incident occurred which aptly illustrates the parties to that opposition. As young Dr. Hullihen sat at his window opening on the street, waiting with small hope, no doubt, for the professional calls that came not, he observed two of the young dandies of the town, one of them a medical student in the office of an established practitioner, coming toward his house; the student carried a chicken cock under his arm, and the manners of both the young men indicated mischief on foot. When they reached his window, they accosted Dr. Hullihen cavalierly with—"Dr. we have brought you a patient this morning, wanting your surgical skill," and the chicken cock with a broken leg was at once exhibited. Hullihen was prompt in seeing the intention to mortify his professional pride, but as promptly and quietly resolved upon the right mode of punishing the offenders. He examined the fracture carefully, and then told the young idlers it was readily within the means of cure; he forthwith set about the operation; splints were very nicely prepared and adapted, the young men acting as assistants. "And now," said the surgeon, "you must leave this patient with me for about ten days, during which time he shall be kept quiet under a barrel in my cellar, and provided with the proper diet; at the end of that time you will please call for him." This was a very unexpected turn of the case to the young dandies, and they left the office of Dr. Hullihen with no intention of ever returning for the poor cock whose leg they had purposely and wickedly broken merely to play a trick upon the doctor. Finding they did not come back in a fortnight, Dr. Hullihen politely notified them of the patient's entire cure and readiness to return home. No attention being paid to this message, he sent back the chicken with a bill of twenty dollars for professional services. The charge was ridiculed, as he expected, and payment refused; but the doctor notified them that he would bring suit, and the matter was accordingly brought before a magistrate. Hullihen made his own simple statement of the facts, and *judgment was given in his favor for the full amount of the bill:* this sum the doctor requested should be given to a charitable institution. By this result of a practical joke, the jokers became the laughing-stock of the whole town, while the man they intended to mortify was advanced in popularity and public estimation.

The general state of dental surgery at the southwest, and indeed anywhere, twenty or twenty-five years ago, leaves one greatly in the dark as to whence Dr. Hullihen could have derived his high standard of practice in that department of surgery. He had read and digested the English works and a few translations from the French then existing, but these afforded very slender capital in the way of example or guidance to a man beginning his professional career in a small Western town. His reliance then, we may safely conclude, was in his own natural abilities, backed by an honest and persevering ambition.

In truth, Hullihen was a man of true genius, and especially gifted in reference to original conceptions whereby to overcome difficulties; he possessed the discriminating mind, the rapid eye, and the cunning hand that act in harmony and combine to produce spontaneous and correct decisions. These qualities, so peculiarly marking the great and successful surgeon, became at once apparent to the sufferer for whose relief Dr. Hullihen was called in; it was not merely when he assumed the task of a bold and difficult operation, but when his earnest, clear and methodical mind applied itself to the examination of a case, there was a faith-inspiring confidence in his own heart permitting no doubt that the very best interests of the patient, and nothing else, would govern the surgeon's rule of action.

Hullihen had a warm and most generous heart—one so full of the best impulses toward his fellow-men that he never found room there for himself, or time to consider his own interests; and yet such was the untiring industry of his active professional life, and the many strong personal attachments he inspired, that grateful and unexpected compensations continually came to his ungrasping hand, to be again scattered by an unselfish liberality.

Dr. Hullihen received the degree of Doctor of Medicine from the Medical Department of Washington College, Baltimore, Maryland; but he had no wish to, and never did practice general medicine. His great success and usefulness appeared in surgical operations, and these were not confined to the town of Wheeling: patients of all classes, confiding in his skill and frank character, came in great numbers from the surrounding country, and it is no exaggeration to state that the value of his services as well as the influence of his reputation eventually came to be considered precious as the common property of Wheeling. Hence, whenever any worthy scheme, whether charitable or political, was to be successfully carrried through, it seemed a necessary preliminary to obtain for it the interest, approbation, and cooperation of Dr. Hullihen. On such occasions—and they were numerous beyond credibility—the object, whatever it might be, was sure to receive the most active indefatigable efforts of which he was capable, and others were soon brought over to it by his generous example no less than by his sound, sensible arguments. Prominent among the good projects of Dr. Hullihen was his great desire to establish a public hospital at Wheeling, where the sick and disabled of every class might find proper attendance while under medical treatment or surgical operations. He well knew that much suffering might be spared by judicious attentions, and that, in many instances, lingering maladies, and even the loss of life itself, was the sad result of bad nursing. To found such a hospital he exerted himself in every direction that seemed practicable: as a member of the city council and otherwise, he endeavored to enlist the city itself in the humane enterprise, but without success. Nor was he able, during several years of effort, to secure any material aid from any quarter. In the mean time his own growing reputation as a surgeon, occulist, and dentist was, as I have said, attracting patients from a distance, and somewhat to meet the exigencies of his private practice, he established an infirmary of his own. The prosperity and increasing importance of this unpretending charity became the cherished object of his daily and devoted attention, and at length he concerted measures with

Bishop Whelan, a gentleman of great energy and practical benevolence, to establish a hospital under the charge of one of the religious orders of the Catholic church. Having secured the aid of the "sisters," with the co-operation of the bishop, he entered upon the work of building up his favorite institution with all the earnestness and devoted zeal which marked his character. A house was purchased by the bishop for the residence of the "sisters," and Dr. Hullihen supplied it with patients at once to the extent of its capacity. This beginning must have been made about seven years ago, and not long afterward (March 12, 1850) a charter was obtained, for a corporation under the name of "The Wheeling Hospital." This corporation is now the owner of a large and commodious building at the north end of the city, a property purchased two years since, and the building has been subsequently much enlarged. At the time of Dr. Hullihen's death it had become the resort of patients from all parts of the country, brought there chiefly by his high reputation and remarkable success. He had not only contributed largely to the prosperity of the institution by his strictly professional exertions, but he was assiduous in his care for its good management in every department. Although assisted by the contributions of some benevolent persons, and by the active exertions and liberal expenditures of the bishop, as well as the gentle charity of the good "sisters," and although sustained by the professional skill of doctor M. H. Houston, the eminent physician of the hospital, Dr. Hullihen was the founder—the life and soul of the enterprise. Its success was assured by the co-operation of others, but it would have been impossible without him. He left it in a flourishing condition, and it is probable that his efforts in connection with it and in his general practice overtasked his physical powers and contributed to bring on his untimely death.

Without pretending to offer full statistics of the operations performed by Dr. Hullihen, it may convey a correct idea both of the range and the extent of his surgery to state that, during the last ten or twelve years of his life, the professional memorandums on his books show that he operated—

| | | | |
|---|---|---|---|
| For Cataract, | about | 200 | times. |
| " Hare-lip, | " | 100 | " |
| " Cleft palate, | " | 50 | " |
| " Cancers, | " | 150 | " |
| " Antrum cases | " | 200 | " |
| " Strabismus, | " | 100 | " |
| Making new noses, | " | 25 | " |
| " new lips, | " | 50 | " |
| " under-jaws, | " | 10 | " |
| General surgery, | " | 200 | " |

Add to these the busy practice of a successful dentist in all the branches of his profession, and we exhibit an extraordinary amount of labor, of energy and usefulness, such as few men have lived to accomplish even in a much greater number of years. But, however great the industry needed to meet the demands upon his skill, and the wear upon his frame, when the truth is known, that nearly one-third of all this labor was the work of charity, we form a just estimate of the tax upon a noble heart and a self-sacrificing nature. It was a rule of his professional life to make no charge whatever for services rendered to clergymen, no matter where resident or of what persuasion: thus, in the department of artificial teeth, Dr. Hullihen supplied in a single year charity-work amounting to about two thousand dollars, at the ordinary rate of charges to others. There is no need of comment upon the liberality displayed on the one side and abuse of it on the other.* An anecdote in this connection seems apropos.

Hullihen had encountered some ungrateful cases that were not a little trying to his patience, when he was applied to by a country gentleman of the pulpit, with very miserable teeth,—one indeed who had only a sad array of decayed fangs. This patient expressed great wonder as to the cause of diseased teeth and their loss when life was scarce half over;—"it was unnatural: it could never have been the design of Providence!" and he wished the doctor would explain the mystery. The general causes that lead to decay and loss of teeth were very carefully gone over by the doctor, and the visitor, with a mind apparently enlightened and satisfied, left the office; but in a day or so the same gentleman returned, and again he wanted the doctor to tell him *why* it was that teeth decayed; why *he* should be thus sorely afflicted so early in life. A little impatient at the evident inattention to his previous explanations, the doctor now said:—"Well, sir, I very recently gave you a *professional* view of the causes of decay in teeth, which you seem not to have understood, so I will now give you a *scriptural* explanation of that misfortune, which you will understand. You are, of course, conversant with the scriptural history of the forbidden-fruit, and the sin of our first parents; you must perceive that when Adam and Eve ate the apple they bit it with their teeth; of course, then, the teeth being the immediate instruments of sinful disobedience, it was right that they should be the greatest sufferers for the offence. Therefore, we may conclude, that whenever a man has very bad teeth early in life, there is an unusual amount of old Adam in his nature!" This original opinion of the cause of decayed teeth was probably better remembered by the patient than the previous scientific one.

Dr. Hullihen published many valuable papers on medical subjects, but they have not been preserved or collected in such a form as to permit a correct reference to their extent. Those that have come to my knowledge are as follows:—

1839—*An Essay on Odontalgia.*

1844—*A Treatise on Hare-Lip and its Treatment.*

1845—*An Essay on Cleft-Palate and its Treatment.*

1846—*An Essay on Abscess of the Jaws and Treatment.*

1849—*Report of a Case of Elongation of the Under-Jaw, with Distortion of the Face and Neck, caused by a Burn, Successfully Treated.*

Dr. Hullihen's correspondence was very extensive, and from his letters, as well as publications, his style may be regarded as clear and energetic, with an earnest warmth, a direct truthfulness, and originality that strongly partook of his character.

He was the inventor of many new forms of instruments of great value to the dentist and the surgeon, but he never took credit to himself for these improvements, allowing the cutlers the free use of what he originated. As a dentist, Dr. Hullihen was eminently judicious and skillful. His elevated

* It is sad to say, those who most deserve such exemption, and for whom it is a pleasure to exercise professional skill, are the very men who are least inclined to accept such favors.

views of practice have been embodied in his own language, when he said:—"*The dentist must carry upward the standard of his profession and plant it upon the broad platform of medical science; he must claim for himself and his profession the same respect and importance awarded to other branches of the healing art, and that, too, upon the same ground,—the ground of a thorough scientific education.*" It is not practicable here to give a history of the various improvements and discoveries contributed to dental surgery by Dr. Hullihen, but I desire, at least, to name, with special interest, his very valuable improvement in the operation of filling teeth when the decay has reached the nerve-cavity. This original device consists in perforating the fang through the gum and alveolar process into the nerve, before packing the metal, and has been particularly described to the profession in the pages of the *Philadelphia Medical Examiner* for Oct. 1852. This successful mode of filling teeth when the nerves are exposed, has justly been termed the *Hullihen Operation*, and has been approvingly and gratefully adopted by the best dentists of our country.

Those who were best acquainted with Dr. Hullihen, and able to appreciate his value, in the profession as well as out of it, will not be satisfied with any other title for him than that of *a great man*. Hence the monument being erected to his memory by the city of Wheeling was justly due to his character and services; it was the natural act of a sorrow-stricken community, and is the more honorable as the spontaneous movement of a grateful people in whose midst the best years of his life had been spent, and is a true expression of the well-earned love and gratitude existing toward him, and realized at the period of his untimely death.

In this brief sketch I am conscious of having done but imperfect justice to the life of a very distinguished man, with whose warm friendship I was honored; but my consolation is, that it has at least been a work of love, the only duty and privilege I could claim after watching by his bedside the last three days of his existence.

Dr. Hullihen's death was indirectly caused, as before intimated, by his great devotion to the duties of the Wheeling hospital: he took a severe cold from sudden exposure after being heated in the operating theatre, bringing on pneumonia and fever that became unmanageable, even by the best medical skill and attention. E. B. G.

